# From MASLD to MELD 3.0: Integrating the Spectrum of Liver Disease Risk Stratification into Primary Care

**DOI:** 10.5152/eurasianjmed.2026.251254

**Published:** 2026-03-05

**Authors:** Pavan Kumar Reddy Kalluru, Nirmal Onteddu, Lourdhu Pragna Reddy Onteddu, Shivani Thota, Apoorva Cherukuri, Hossein Rajali, Kalyan Naik Gugulothu

**Affiliations:** 1 Department of Internal Medicine, West Anaheim Medical Center, Anaheim, California, USA; 2 Department of Internal Medicine, University of Florida College of Medicine, Jacksonville, USA; 3 Department of Health Sciences, University of Ottawa, Ottawa, Canada; 4 Department of Gastroenterology, Kamineni Institute of Medical Sciences, Narkatpalli, India; 5 Department of Physical Diagnosis, American University of Anguilla School of Medicine, Anguilla

**Keywords:** Fatty liver, metabolic syndrome, non-alcoholic steatohepatitis, liver cirrhosis, primary health care

## Abstract

In 2023, key advancements in liver disease were introduced into practice. Metabolic dysfunction-associated steatotic liver disease (MASLD) replaces nonalcoholic fatty liver disease (NAFLD). Model for end-stage liver disease (MELD) 3.0 replaced MELD-sodium. The prevalence of MASLD among adults is approximately 38%, and it is anticipated to rise by more than 55% by 2040. The economic burden in the USA related to MASLD exceeds $100 billion. If unrecognized, 20%-30% of adults with MASLD advance to MASH, cirrhosis of the liver, and may develop hepatocellular carcinoma. In the last 30 years, deaths related to cirrhosis have increased. As clinical approaches to screening are enhanced, the primary care physicians need to be indoctrinated. By recognizing risk factors, primary care providers can reduce the national disease burden and expedite interventions to improve patient outcomes. This manuscript aims to increase awareness of the availability of evolving knowledge necessary to minimize gatekeeping in the liver disease care path.

**Main Points**

- Parallel paradigm shifts in liver disease: The transition from nonalcoholic fatty liver disease to metabolic dysfunction–associated steatotic liver disease (MASLD) and from model for end-stage liver disease-sodium (MELD-Na) to MELD 3.0 represent 2 ends of a unified framework for precision risk stratification across the liver disease spectrum.
- Metabolic dysfunction–associated steatotic liver disease in primary care: Early identification of MASLD using non-invasive fibrosis tools (e.g., Fibrosis-4 Index, NAFLD Fibrosis Score, and FibroScan) enables primary care physicians to triage patients based on fibrosis stage and metabolic risk.
- Model for end-stage liver disease 3.0 for advanced disease: The updated MELD 3.0 formula enhances prediction of short-term mortality and corrects gender disparities in liver transplant prioritization.
- Integrating risk stratification across care levels: Linking MASLD screening in primary care with MELD 3.0–based assessment in specialty settings supports timely referral, reduces waitlist inequities, and improves outcomes.
- Unified care pathway: A continuum-based approach—from metabolic dysfunction detection to transplant risk assessment—can streamline liver disease management and reduce national disease burden.

## Introduction

In 1980, the term “non-alcoholic” was first used to describe the histologic liver changes resulting from excess fat accumulation in the absence of significant alcohol intake, referred to as non-alcoholic steatohepatitis.^1^ Later, the broader term non-alcoholic fatty liver disease (NAFLD) was introduced. However, there have been ongoing discussions about adopting a more appropriate nomenclature since then.^2^ In 2020, a new term, metabolic dysfunction–associated fatty liver disease, was proposed to replace NAFLD and was widely accepted.^3^ In 2023, the nomenclature was further refined into metabolic dysfunction–associated steatotic liver disease (MASLD) through a multi-society Delphi consensus statement.^4^ In the same statement, metabolic dysfunction and alcohol-related liver disease (MetALD) was also introduced.^5^ Along with this name change, researchers introduced a new diagnostic criterion, which is gaining increasing acceptance. The transition from NAFLD to MASLD and from model for end-stage liver disease-sodium (MELD-Na) to MELD 3.0 represents 2 ends of a unified paradigm shift: precision risk stratification across the entire spectrum of liver disease. While MASLD emphasizes early metabolic recognition in primary care, MELD 3.0 refines end-stage prognosis and equity in transplantation. Together, these frameworks underscore the need for integrated liver care pathways spanning from primary detection to tertiary referral.

## Discussion

### Diagnostic Criteria for Metabolic Dysfunction-Associated Steatotic Liver Disease

**Figure 1.**
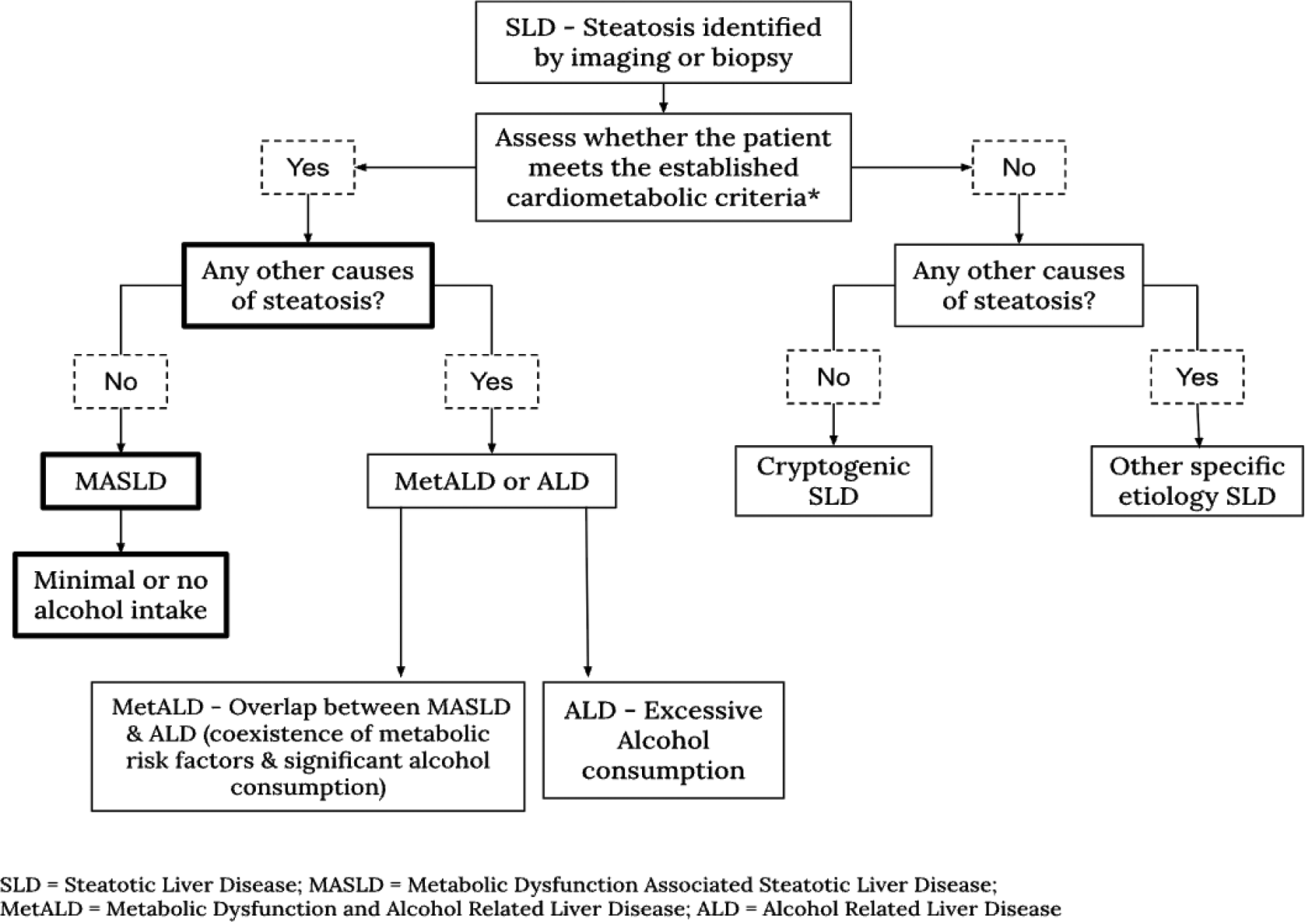
Differentiation and definitions of liver disease terminology.

Metabolic dysfunction-associated steatotic liver disease is defined by hepatic steatosis (≥5% fat in liver cells) and metabolic dysfunction, as determined by cardiometabolic criteria*. The diagnosis requires ruling out other causes of liver fat accumulation. While minimal alcohol intake is allowed, significant alcohol consumption would classify the condition as MetALD or ALD instead.^4^ Figure 1 illustrates this classification.

*Cardiometabolic criteria: At least 1 out of the following:

1. BMI of 25 kg/m^2^ or greater (cut off in Asian population is 23 kg/m^2^) or waist circumference of more than 94 cm in males and 80 cm in females or ethnicity adjusted equivalent.
2. Fasting serum glucose ≥ 5.6 mmol/L or 2-hour post-load glucose levels ≥ 7.8 mmol/L or HbA1c (glycated hemoglobin) ≥ 5.7% or type 2 diabetes or treatment for type 2 diabetes.
3. Blood pressure ≥130/85 mmHg or use of antihypertensive medications.
4. Triglycerides ≥ 1.7 mmol/L or specific lipid-lowering treatment.
5. HDL-cholesterol ≤ 1.0 mmol/L in males and ≤ 1.3 mmol/L in females or specific lipid-lowering treatment.

### Epidemiology

Excess liver fat (in over 5% of liver cells) is a hallmark of MASLD (a term that will be used throughout the article when referring to older terminology, at least most of the time), which is becoming increasingly common worldwide. Currently, MASLD affects over 30% of the global adult population, and its prevalence is steadily rising.^5^ In addition to placing a significant burden on healthcare costs, MASLD is associated with higher rates of mortality from both cardiovascular and liver-related causes.^6^ According to the Global Burden of Disease study, MASLD is a substantial and the fastest-escalating global contributor of health impact from chronic liver disease consequences, such as cirrhosis and hepatocellular carcinoma (HCC).^7^ In the USA and some Western nations, MASLD has emerged as the leading cause of liver transplants for patients with HCC in recent years. It is also the second most prevalent reason for transplantation in individuals without HCC.^5^^,8^ Similar trends are observed in the latest United States United Network for Organ Sharing (UNOS) data.^9^ This surge is majorly driven by the rising prevalence of obesity and type 2 diabetes mellitus.^10^ Therefore, referring to MASLD as an ongoing or emerging pandemic is not wrong. Additionally, it is essential to note in this section that the incidence and prevalence of MASLD are higher in males compared to females.^11^

### Primary Care’s Role in Metabolic Dysfunction–Associated Steatotic Liver Disease: Where Does It Stand?

Metabolic dysfunction–associated steatotic liver disease/NAFLD progresses through several stages (steatosis → steatohepatitis → fibrosis → cirrhosis → liver failure/hepatocellular carcinoma), potentially leading to the need for liver transplantation. It’s important to note that disease progression is heterogeneous, with some patients advancing more rapidly than others. Factors such as the presence of diabetes, the severity of histological features, and changes in specific serum markers may influence the rate of progression.^12^ Several tools are available for assessing liver disease severity in MASLD and cirrhosis, aiding in risk stratification, fibrosis assessment, and prognosis prediction. The most common tools used in primary care for early MASLD detection include the NAFLD Fibrosis Score (NFS), Fibrosis-4 Index (FIB-4), and transient elastography/FibroScan. According to a study, the non-invasive FIB-4 has demonstrated exceptional efficacy in ruling out advanced fibrosis, with a negative predictive value (NPV) of 95.7%.^13^ With an area under the receiver operating curve (AUROC) of 0.88, it is beneficial for identifying advanced fibrosis. The NFS also showed good diagnostic accuracy for advanced fibrosis (NPV 93.0% and AUROC 0.86).^13^ Although values may vary with different studies, FIB-4 and NFS have consistently shown clinical utility. Transient elastography, an imaging-based technique, evaluates fibrosis (liver stiffness) and steatosis (controlled attenuation parameter).^14^ The limitation is the availability of equipment for primary care. Meanwhile, FIB-4 and NFS are simple, non-proprietary scores, easily calculated using routine clinical and laboratory parameters.^15^ Other less commonly used tools include the Enhanced Liver Fibrosis (ELF) Test and AST to Platelet Ratio Index. The particular clinical setting and equipment availability often influence the tool choice.

In general, non-invasive tests such as FIB-4 and the NAFLD Fibrosis Score are commonly utilized as practical first-line tools for identifying individuals at high risk for NAFLD. These straightforward scoring systems integrate clinical risk factors for fibrosis, such as age and diabetes, with regular biochemical tests. Low scores from these tests provide significant negative predictive values, helping to rule out severe fibrosis. Individuals with high or indeterminate FIB-4/NFS scores require further evaluation using second-line biomarkers, such as liver stiffness measurements or the serum ELF test. They may require referral to a hepatology clinic for further assessment of liver disease severity.^16^ Metabolic dysfunction–associated steatotic liver disease is usually asymptomatic and often presents with normal or mildly elevated liver enzymes, leading primary care physicians (PCPs) to underestimate its prevalence. This likely results in a substantial proportion of individuals remaining undiagnosed and therefore untreated. As the first point of contact in the healthcare system, PCPs play a critical role in coordinating care and referring patients to specialists or hospital services. Therefore, it is essential to empower PCPs to confidently diagnose and manage MASLD, especially since a large proportion of patients have low-risk disease. However, a key challenge is that PCPs are often unfamiliar with the full clinical spectrum of MASLD and the use of fibrosis biomarkers and diagnostic algorithms.^17^ Additionally, determining the right time to refer patients to specialists such as gastroenterologists or hepatologists can be difficult. Table 1 compares different liver disease assessment tools.

### Evolution of Model for End-Stage Liver Disease: From Its Origins to Model for End-Stage Liver Disease 3.0

The Mayo Clinic introduced the MELD score in the 2000s as a predictive tool for assessing 90-day mortality in patients undergoing Transjugular Intrahepatic Portosystemic Shunt (TIPS).^25^ In 2002, it replaced the Child-Pugh (CP) score as the primary tool used by the UNOS to prioritize liver transplant candidates based on medical urgency rather than waiting time. The CP score included bilirubin (mg/dL), albumin (g/dL), International Normalized Ratio (INR), ascites, and hepatic encephalopathy. Unlike the subjective elements of the CP score (hepatic encephalopathy and abdominal ascites), MELD provided an objective and reproducible method using laboratory values—serum bilirubin, INR, and serum creatinine—for assessing short-term mortality risk, significantly improving fairness in organ allocation and reducing waitlist mortality.^23^ Patient outcomes were also influenced by other variables, such as serum sodium, albumin, lactate, age, sex, ascites, encephalopathy, and glomerular filtration rate. Model for end-stage liver disease’s prognostic accuracy was inferior, particularly for serum sodium,^26^ and the need for refinement became evident.

Model for end-stage liver disease-Na was introduced to address some of these limitations by incorporating serum sodium to improve mortality prediction. In 2016, MELD-Na became the standard score for the UNOS/Organ Procurement and Transplantation Network (OPTN).^27^ It was created during a period when hepatitis C was the primary reason for liver transplantation. With the decline in hepatitis C prevalence and the rise of MASLD and MetALD, MELD-Na became less predictive.^28^ Model for end-stage liver disease-Na capped serum creatinine at 4 mg/dL (0.354 mmol/L), meaning patients with higher creatinine levels were assigned a similar mortality risk, regardless of dialysis dependence.^29^ It also did not account for gender differences. Women who have lower muscle mass and thus lower creatinine levels were systematically disadvantaged on the transplant waitlist.^30^ Several variations of the MELD score were subsequently developed to include additional variables, notably MELD-Lactate,^31^ MELD-Plus,^32^ and MELD-GRAIL-Na.^33^ Proposed in 2017, MELD-Lactate incorporated lactate to predict mortality but was adapted for critically ill patients.^31^ Model for end-stage liver disease-Plus was proposed in 2018 by including additional variables, such as albumin, in the original MELD score; however, its complexity limited its widespread adoption.^32^ Model for end-stage liver disease-GRAIL-Na was published in 2019 and included more variables, such as racial variation alongside sodium, but had limited clinical utility.^33^ Despite these advancements, none of these alternative models were adopted as the standard for UNOS/OPTN. Model for end-stage liver disease-Na remained the standard score until 2023, when MELD 3.0 was adopted as the new transplant allocation model.^34^

### Model for End-Stage Liver Disease 3.0: Breaking Down the New Scoring System

To address the limitations of earlier models, MELD 3.0 was first published in 2021^34^ and standardized by UNOS/OPTN in 2023. This updated version included sex as a variable to correct gender disparities, modified the weightings of creatinine and sodium, and introduced albumin as an additional variable.^34^ Albumin is a crucial indicator of liver pathologies, and its inclusion in the score resulted in more accurate risk prediction than previous models.^35^ The gender-based adjustment of the score resulted in women having slightly higher scores, addressing the traditional disadvantage women faced in transplant allocation.^36^ Model for end-stage liver disease 3.0 retained the sodium variable, influencing mortality in end-stage liver disease. Adjustments to the weightings of creatinine and sodium have improved the accuracy of early (90-day/3-month) mortality risk, particularly in women and individuals with lower creatinine levels.^37^ Model for end-stage liver disease 3.0 demonstrates enhanced accuracy in predicting 3-month mortality compared to MELD-Na, as reflected by a higher concordance statistic (0.869 vs. 0.862).^34^ A temporal validation study of transplant subjects from 2019 revealed that MELD 3.0 recategorized 8.8% of deceased waitlisted subjects into higher MELD categories, predominantly among females.^37^ Overall, MELD 3.0 has contributed to a fairer organ allocation system by prioritizing the sickest patients for liver transplantation and improving the accuracy of 3-month mortality prediction.

MELD 3.0 Formula:

MELD = 9.57×log_c_(creatinine) + 3.78×log_c_(bilirubin) + 11.20×log_c_(INR) + 6.43

MELD 3.0 = 1.33 (if female) + 4.56×log_c_(bilirubin) + 0.82×(137 - Na) − 0.24×(137 - Na)×log_c_(bilirubin) + 9.09×log_c_(INR) + 11.14×log_c_(creatinine) + 1.85×(3.5 - albumin) − 1.83×(3.5 -albumin)×log (creatinine) + 6

These algorithms will yield MELD scores, which will be rounded to the closest whole number. Score ranges for MELD 3.0: 06-40. See Table 2 for units of measurement and caps of variables. In MELD and MELD-Na, serum creatinine was noted as 4 mg/dL (0.354 mmol/L) if the patient had 2 or more dialysis sessions in the previous week. In MELD 3.0, serum creatinine for patients on dialysis has been modified to an upper limit of 3 mg/dL (0.265 mmol/L) to avoid overestimating risk.^25^^,^^34^ This advocates for transplant access for women and possibly reducing waitlist mortality.

Limitations of MELD 3.0:

1. Although MELD 3.0 adjusted the model for the female gender, there is still ongoing debate about whether this adjustment fully nullifies the gender inequalities in organ allocation.^38^ This adjustment may be over-corrected for specific subgroups, particularly younger women with preserved renal function.
2. The model lacks dynamic adjustment for acute kidney injury and chronic kidney disease.
3. The model also lacks input on the implications of albumin infusions in patients with low MELD 3.0 scores.
4. The upper limit for creatinine was retained in MELD 3.0 and was reduced by one unit compared to the original MELD and MELD-Na scores.
5. Recent research indicates that for individuals with alcohol-related liver disease, MELD 3.0 is not always superior to either MELD or MELD-Na.^39^

### Integrating Liver Disease Risk Stratification into Primary Care

Advanced fibrosis (stage 3) and cirrhosis (stage 4) are the strongest predictors of mortality and liver-related complications in MASLD. These patients require ongoing surveillance for HCC and the risk of liver decompensation. Those without severe fibrosis can be managed in primary care, emphasizing reducing cardiovascular risk and monitoring disease progression.^40^ Metabolic dysfunction–associated steatotic liver disease increases cardiovascular risk through several interconnected mechanisms, including metabolic dysfunction (insulin resistance, dyslipidemia), inflammatory processes, vascular effects, and cardiac structural changes.^41^ Management approaches to MASLD among PCPs vary considerably; some actively assist patients in setting dietary and physical activity goals, while others provide general recommendations or refer patients to a specialist and/or dietitian for lifestyle management. Optimizing the timing and frequency of specialist referrals is critical to balancing healthcare resource constraints while ensuring optimal patient outcomes. Informal surveys indicate that the average wait time for a gastroenterology appointment is approximately 50 days or more. For hepatology, 1 study reported an average wait time of 68 days (±55 days) for an initial clinic visit.^42^ Model for end-stage liver disease 3.0 score is a mathematical model with the latest iteration to assess short-term mortality risk and prioritize liver transplant in patients with advanced chronic liver disease. Understanding MELD 3.0 empowers internists to advocate for timely referral to specialists, i.e., at the time of clinical decompensation of chronic liver disease or when the MELD score exceeds 15. Original MELD and MELD-NA scores are used to predict survival in TIPS placement, prognostic index in alcohol-related hepatitis, and survival and mortality risk in non-transplant candidates; however, the utility of MELD 3.0 requires further investigation.

## Conclusion

Steatotic liver disease represents a major global health challenge, and its early recognition in primary care provides a critical window to prevent progression to advanced liver disease. By incorporating metabolic risk profiling and noninvasive assessment tools, PCPs can identify patients with advanced fibrosis (F3-F4), thereby substantially reducing long-term complications such as cirrhosis and hepatocellular carcinoma. Furthermore, integrating MELD 3.0 into clinical workflows allows PCPs to enhance risk stratification and identify high-risk individuals (MELD scores > 15) promptly. This enhanced identification bridges the gap between primary and specialty care, facilitates timely consideration for interventions like liver transplant (which offers a survival benefit in advanced cirrhosis or hepatocellular carcinoma), and ultimately addresses the growing burden of liver-related morbidity and mortality.

## Figures and Tables

**Table 1.** Comparison of Liver Disease Assessment Tools

Tool	Primary Use	Parameters	Pros	Cons
Fibrosis-4 Index (FIB-4)	Fibrosis assessment	Age, AST, ALT, platelets	Simple, widely available, and good for ruling out fibrosis.^13^	Less accurate in intermediate values or borderline values.^18^
NAFLD Fibrosis Score (NFS)	Fibrosis assessment	Age, BMI, diabetes, AST/ALT, platelets, albumin	Good for advanced fibrosis in MASLD.^13^	Inaccurate in young patients.^19^ Requires more variables than FIB-4.
AST to Platelet Ratio Index (APRI)	Fibrosis assessment	AST, platelet count	Simple, available in routine labs.	Less accurate in early fibrosis.^20^
Enhanced Liver Fibrosis (ELF) Test	Fibrosis assessment	Hyaluronic acid, TIMP-1, PIIINP	More accurate than FIB-4/NFS for advanced fibrosis.^21^	Needs specialized lab testing.^21^
FibroScan	Fibrosis and cirrhosis staging	Liver stiffness measurement	Non-invasive, widely used in hepatology clinics.^22^	Requires special equipment and trained personnel.^22^
Child-Pugh (CP) score	Cirrhosis prognosis	Albumin, bilirubin, INR, ascites, encephalopathy	Simple, widely used.	Subjective scoring of ascites/encephalopathy.^23^
Albumin-Bilirubin (ALBI) score	Liver function	Albumin, bilirubin	Simple, objective alternative to Child-Pugh.	Uses only 2 components (albumin and bilirubin), which may not capture the full complexity of liver disease.^24^
MELD 3.0	Cirrhosis severity and prognosis	Bilirubin, creatinine, INR, sodium, Alb, sex	More accurate than MELD-Na, good for MASLD patients.	Not useful for early-stage disease.

AST, aspartate aminotransferase; ALT, alanine aminotransferase; BMI, body mass index; MASLD, metabolic dysfunction–associated steatotic liver disease; MELD, model for end-stage liver disease; NAFLD, nonalcoholic fatty liver disease;TIMP-1, tissue inhibitor of metalloproteinases-1; PIIINP, procollagen III N-terminal propeptide; INR, International Normalized Ratio.

**Table 2.** Units of Measurement and Caps for MELD 3.0, MELD-Na, and MELD Variables

**Factor**		**MELD 3.0**	**MELD-Na**	**MELD**
Creatinine (mg/dL)	Lower cap	1.0	1.0	1.0
	Upper cap	3.0	4.0	4.0

INR	Lower cap	1.0	1.0	1.0
	Upper cap	—	—	—

Bilirubin (mg/dL)	Lower cap	1.0	1.0	1.0
	Upper cap	—	—	—

Sodium range (mEq/L)	Lower cap	125	125	Not included
	Upper cap	137	137	

Albumin range (g/dL)	Lower cap	1.5	Not included	Not included
	Upper cap	3.5		

Gender adjustment	—	+1.33 if female	Not included	Not included
Score limits	—	06-40	06-40	06-40

MELD, model for end-stage liver disease.

## Data Availability

The data that support the findings of this study are available on request from the corresponding author.
